# Multidisciplinary Oral Rehabilitation in a Patient With Dental Anomaly: A Case Report With 18-Month Follow-Up

**DOI:** 10.1155/2025/5565892

**Published:** 2025-02-24

**Authors:** Rodrigo Silveira Tosta Figueiredo, Thiago de Almeida Prado Naves Carneiro, Nayara Rodrigues Nascimento Oliveira Tavares, Ângela Braga Caixeta, Michelle Pereira Costa Mundim Soares, Paulo Vinícius Soares

**Affiliations:** ^1^School of Dentistry, Federal University of Uberlândia, Uberlândia, Minas Gerais, Brazil; ^2^Implantologist and Oral Surgeon, Federal University of Uberlândia, Uberlândia, Minas Gerais, Brazil; ^3^Department of Endodontics, Anhanguera School of Dentistry, Uberlândia, Minas Gerais, Brazil; ^4^School of Dentistry, Pontifical Catholic University, Belo Horizonte, Minas Gerais, Brazil; ^5^Periodontist and Orthodontist, Federal University of Uberlândia, Uberlândia, Minas Gerais, Brazil; ^6^Graduation Program of Operative Dentistry, School of Dentistry, São Leopoldo Mandic, Campinas, São Paulo, Brazil

**Keywords:** dental anomaly, multidisciplinary, oral rehabilitation

## Abstract

The aim of this case report is to sequentially demonstrate the aesthetic and functional rehabilitation of a smile with a dental fusion anomaly. Endodontic retreatment was performed followed by periradicular surgery on Tooth 12. Periodontal intervention through gingivoplasty and aesthetic rehabilitation with composite resin veneers was performed on Teeth 13 to 23. With a clinical and radiographic follow-up of 18 months, the success of multidisciplinary oral rehabilitation involving endodontics, oral and maxillofacial surgery, periodontics, and restorative dentistry is observed, restoring function, aesthetic satisfaction, and quality of life to the patient.

## 1. Introduction

When discussing quality of life, social well-being, professional success, and relationships, dental alterations can be inferred to play a modulating role. Regardless of whether or not your characteristics are fatal, they can alter the psychological dimension of the patient. Therefore, there are various methods to measure oral health–related quality of life (OHRQoL), including through social indicators, questionnaires, and self-classifications [1].

There are numerous dental alterations that go beyond aesthetics, which are subjective and dependent on how the patient, together with the dentist, perceives them [2]. In society, there are standards that are considered contrary to the aesthetics of a smile, such as dental crowding, gingival smile, diastema, and hypodontia [3].

Developmental disorders of the dental organ are a type of alteration that affects the patient's quality of life. Dental fusion is an anomaly in which dental germs from adjacent teeth unite during development, forming a significantly enlarged clinical crown. Its etiology is still uncertain, but there are several hypotheses for its appearance [4].

Identifying dental anomalies during clinical care is essential, especially in cases where fusion with supernumerary teeth has occurred. Supernumerary teeth are also considered a developmental anomaly and are those teeth in excess of the normal number of permanent or deciduous dentitions. With a frequency of occurrence ranging from 0.3% to 3.8% of fused teeth in the population, treatment is highly effective when combined in a multidisciplinary manner [5].

Therefore, this article is aimed at presenting a case report to sequentially demonstrate the aesthetic and functional rehabilitation of a smile with a dental fusion anomaly. The presented planning can be used, in part, in a personalized manner for the dental surgeon's clinical routine, according to the needs and characteristics of each patient.

## 2. Case Report

**Figure 1:**
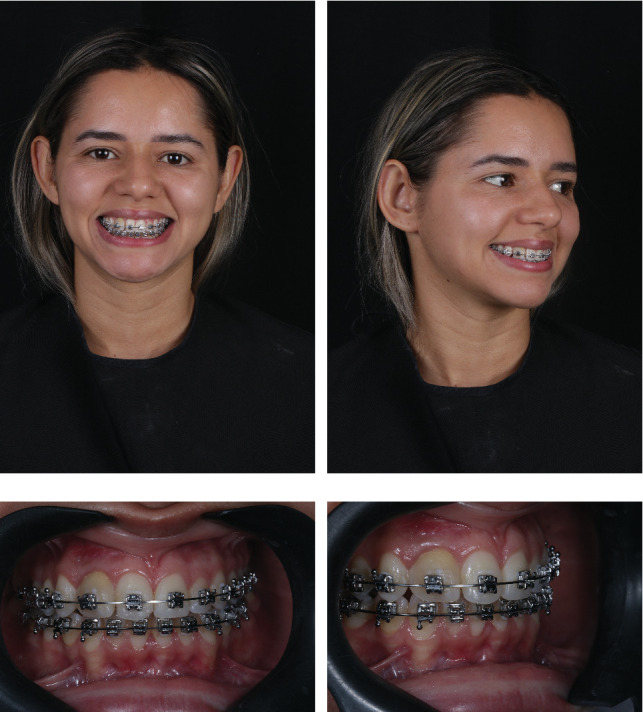
(a) Extraoral photograph of the smile. (b) Extraoral right lateral photograph. (c) Initial frontal view of the smile. (d) Right lateral view of the smile.

**Figure 2:**
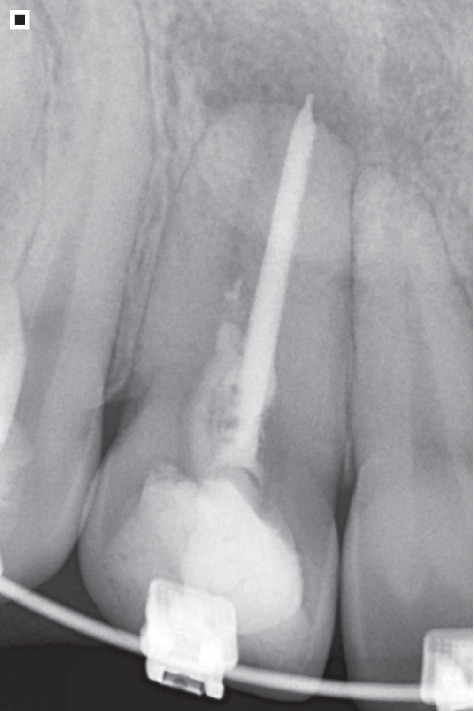
Periapical radiograph of Tooth 12.

A young female patient, 28 years, in the final stages of orthodontic treatment, presented to the dental clinic with complaints about the aesthetics of the smile (Figure 1). During the clinical and radiographic examination (Figure 2), Dental Element 12 with a dental fusion anomaly, possibly with a supernumerary tooth, was observed, where there are clinically joined crowns and radiographically independent root canals. Thus, the patient exhibits an anomaly in both shape and previously in number, in which during the development of the dental organ, fusion occurred with the Dental Element 12.

**Figure 3:**
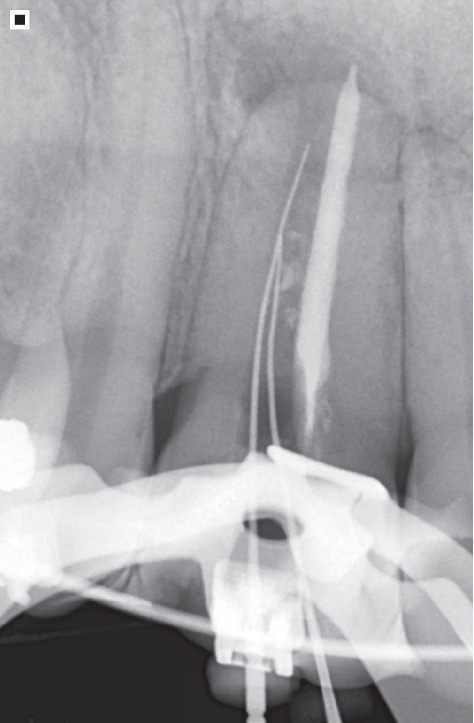
Periapical radiograph, where it can be observed using an endodontic file that there is an untreated root canal. Additionally, a radiolucent area is observed at the root apex.

It is important to note that the differential diagnosis involves a gemination anomaly, in which the dental bud attempts to divide but fails. Consequently, the crown undergoes invagination and is partially or completely divided, extending to the root, forming two similar structures. Radiographically, the observations include adjacent joined crowns, an enlarged pulp chamber, and a shared root canal with bifurcation in the apical region [5–7]. Therefore, considering the presence of two root canals in the present case, the diagnostic hypothesis leans towards fusion anomaly. Furthermore, continuing the anamnesis and clinical examination, the patient reported painful symptoms, despite the endodontic treatment previously performed on the involved tooth. In particular, the periapical radiograph revealed a filling of only one root canal and a radiolucent aspect at the root apex (Figure 3).

**Figure 4:**
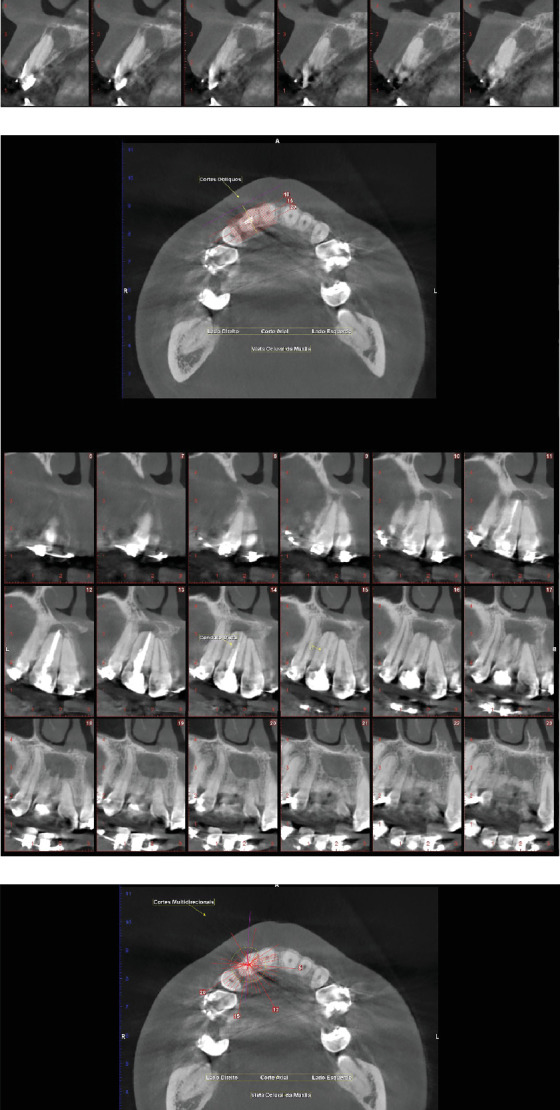
(a–c) Computed tomography showing an overall view of the lesion, canal anatomy, mineralized tissue isthmus, and bifurcation in the third apical. Transaxial, oblique, and multidirectional cuts of the region of Tooth 12 for visualization of the bone and dental structures. Tooth 12 shows a longitudinal hypodense (radiolucent) image on the distal surface of the root, suggestive of an untreated distal root canal, as well as a unilocular, expansive hypodense lesion located in the apical region.

Given the complexity of the case, a computed tomography (CT) of the region in question was performed for a three-dimensional (3D) analysis with half-millimeter thick slices and a half-millimeter space between them (Figure 4). The scan revealed primarily an extensive, well-defined, and hypodense unilocular lesion. Thus, the diagnostic hypothesis is a radicular cyst, characterized by a pathological cavity externally lined by connective tissue and internally covered by the epithelium, filled with semiliquid or liquid content [8].

**Figure 5:**
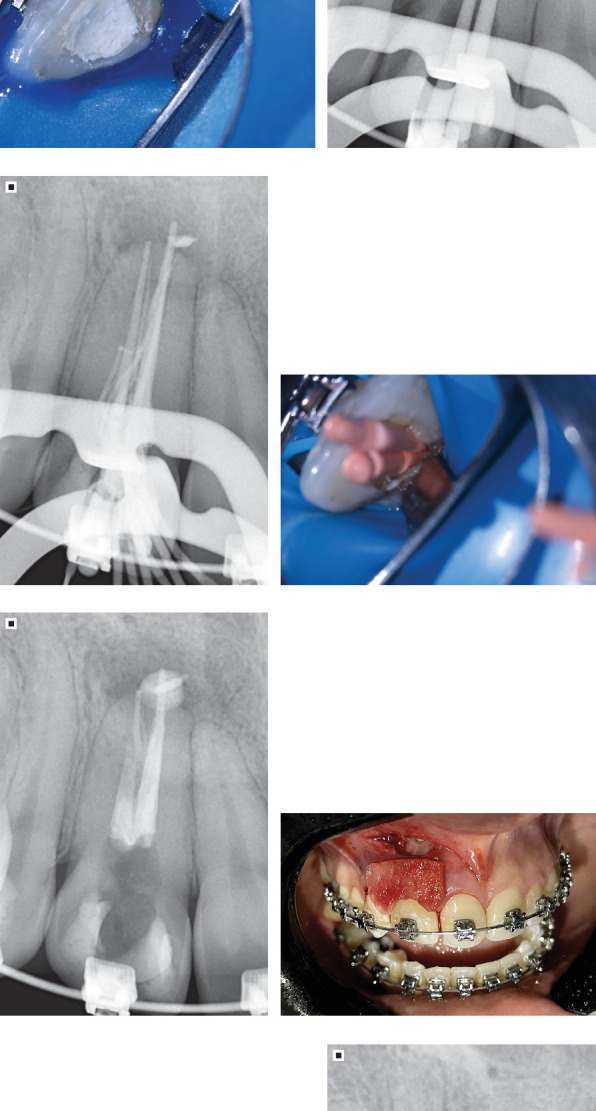
(a) Occlusal view, initial aspect after coronary access. (b) Radiographic aspect of Tooth 12 during removal of obturation material and canal decontamination process. (c) Occlusal view, completion of the first clinical session in which gutta-percha removal and root canal instrumentation were performed, showing restoration with provisional zinc oxide and eugenol cement (IRM, Dentsply Sirona) during the 7-day intervals between sessions in which intracanal medication was applied. (d) Periapical radiograph of the gutta-percha cone fitting. (e) Periapical radiograph prior to filling the root canal. (f) Occlusal view during the final clinical session where instrumentation was completed and root canal filling performed. (g) Periapical radiograph showing the final aspect after endodontic retreatment. (h) View of the dermal acellular matrix before stabilization with sutures. (i) Final presentation after stabilization of the dermal acellular matrix using simple and simple and suspension sutures. (j) Periapical radiograph showing the final aspect after periradicular surgery due to the radicular cyst, diagnosis confirmed postoperatively by oral and maxillofacial pathology laboratory. (k) Occlusal view after definitive restoration of coronary access.

For the patient's aesthetic and functional rehabilitation plan, the initial steps included endodontic retreat coupled with periradicular surgery. Following anesthesia and absolute isolation, coronary access was performed to initiate endodontic retreatment. Subsequently, the second untreated canal was identified. Due to the high anatomical complexity of the canals, decontamination and removal of filling material were performed using the R2 Flatsonic gold ultrasonic insert (Helse Ultrasonic, Brazil), enabling contact with canal walls and optimizing material removal. This was accompanied by the agitation of a 2.5% sodium hypochlorite irrigating solution using the E1 Irrisonic ultrasonic tip (Helse Ultrasonic, Brazil) to improve environmental disinfection and removal of the smear layer [9]. Complete canal decontamination was achieved over three sessions spaced 7 days apart, using Bio-C Temp bioceramic intracanal medication (Angelus, Brazil). Subsequently, the canal filling was performed using multiple gutta-percha cones, with mechanical thermocompaction according to the McSpadden technique to ensure effective 3D sealing [10]. Bio-C sealer cement (Angelus, Brazil) was used due to its physicochemical properties that promote local bacterial neutralization, improving the predictability of a successful endodontic retreat [11]. An apical overflow of 2-mm gutta-percha was designed to enhance apical sealing, facilitating subsequent endodontic microsurgery. Due to the extent and irregularity of the lesion in the apical canal system, apicectomy surgery and retrofilling with Bio-C Repair bioceramic repair cement (Angelus, Brazil) were performed 7 days after filling to enhance treatment success and longevity [12, 13]. The access of the apicectomy involved a semilunar incision in the apical region using a 15c blade, slightly below the lesion, allowing direct access. Following access, granulation tissue curettage was performed, and apicectomy was performed using a 701 long-shaft bur (Prima Dental by Angelus, Brazil) and straight piece on an electric motor (Bien-Air, Switzerland). Subsequent apical plasty employed diamond tips attached to an ultrasonic device (Piezos, CVDentus). The use of an acellular dermal matrix was proposed as an autogenous graft substitute. Mucoderm matrix, a porcine xenogeneic acellular dermal matrix, was selected for its proliferative and supportive properties, promoting rapid anastomosis and subsequent keratinized tissue transformation. The matrix, which was easily handled and inserted, required prior hydration in a saline solution or blood. Subepithelial positioning was achieved through mucoperiosteal detachment and tunneling, with matrix stabilization using techsuture blue nylon 6.0 sutures. Despite higher costs, the advantages of these biomaterials include the elimination of a second surgical site, the reduction of surgical time, and postoperative morbidity (Figure 5).

**Figure 6:**
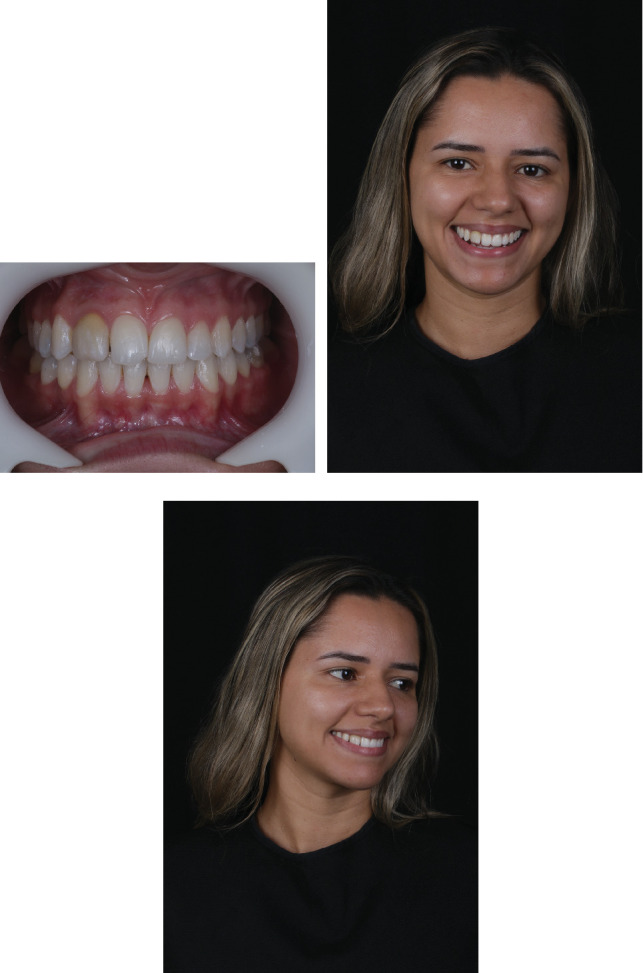
(a) Frontal view of the smile after removal of orthodontic appliances. (b) Extraoral photograph of the smile. (c) Extraoral lateral right view of the smile.

**Figure 7:**
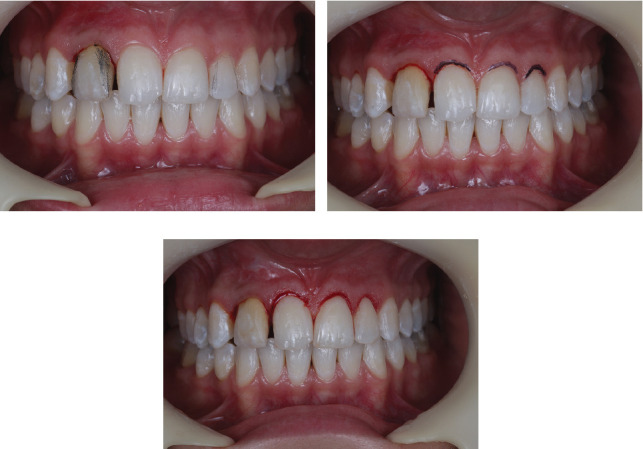
(a) Demarcation with a mechanical pencil of the interproximal wear region of Tooth 12, using a 2200 diamond bur (Prima Dental by Angelus, Brazil) with an electric motor at 100,000 rotations per minute (Bien-Air, Switzerland). (b) Frontal buccal view, with demarcation of the gingival contour of the operative area, showing the amount of tissue to be removed. (c) Immediate final aspect of gingivoplasty surgery.

As the patient had completed orthodontic treatment, the braces were removed in the original clinic (Figure 6), and esthetic treatment was initiated that combined periodontics and dentistry. Initially, gingivoplasty was performed in the upper arch using a high-power laser (Gemini, Ultradent) for precise tissue cutting. However, prior to periodontal surgical procedures, interproximal wear of Tooth 12 was performed to assist in surgical demarcation of the new gingival zenith, according to homologous tooth anatomy (Figure 7). The gingivoplasty procedure was performed due to the presence of a lateral incisor with a dental anomaly, which has a higher gingival margin, resembling a central incisor. This also created an asymmetry with the height of the gingival contour of the corresponding lateral incisor. Therefore, to mask this situation, the gingivoplasty procedure involving the central and lateral incisors was planned.

**Figure 8:**
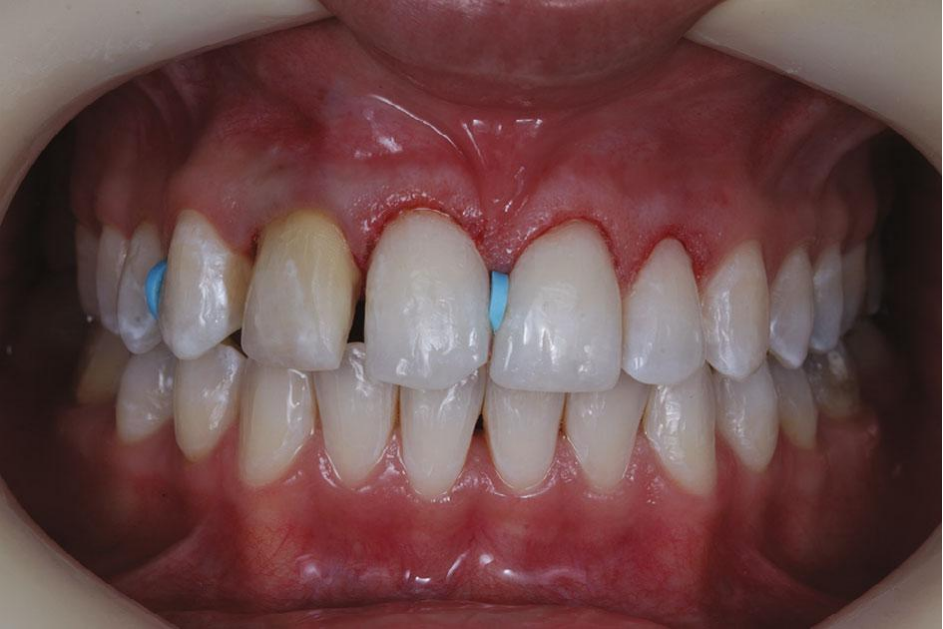
Frontal buccal view after gingivoplasty surgery, with subsequent placement of orthodontic spacers.

**Figure 9:**
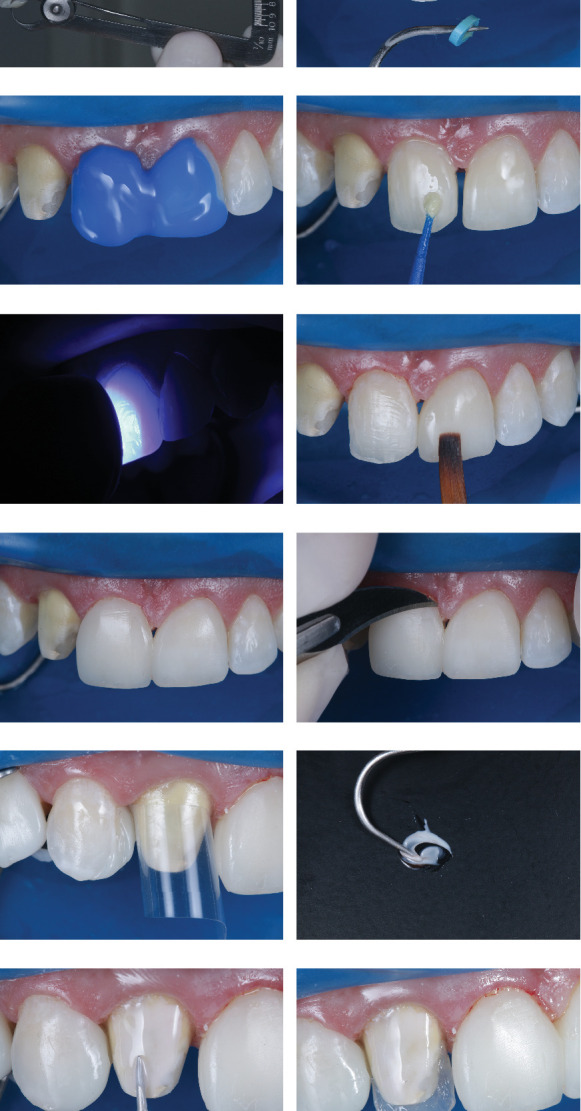
(a) Frontal buccal view, showing gingival health after 7 days of postoperative recovery and successful use of orthodontic spacers. (b) Buccal and palatal wear using a 2135-diamond bur (Prima dental by Angelus, Brazil). (c) Final aspect of the tooth after preparation, creating sufficient working space for anatomical reconstruction of the lateral. (d) Chromatic restoration trial for aesthetic approval by the patient and as a guide for work with FORMA WB resin (Ultradent, United States). (e) Use of a specimeter, showing the restoration trial veneer with only 1-mm thickness. (f) Removal of orthodontic spacers and isolation for restoration of the central incisors. (g) Application of 37% phosphoric acid (Ultra-Etch, Ultradent) for 30 s. (h) Application of universal adhesive (Peak, Ultradent). (i) Photoactivation of the universal adhesive for 40 s (Grand VALO, Ultradent). (j) Application of Transcend UB composite resin (Ultradent, United States) and final shape using wetting resin composite modeling brush (Ultradent, United States). (k) Final aspect after completion of composite resin veneers on Teeth 11 and 21. (l) Removal of excess composite resin in the cervical region using a no. 12 blade. (m) Use of polyester strip for the addition of Transcend A1D composite resin (Ultradent, United States) to the mesial of Tooth 13. (n) Application of opaquer (FinalTouch, VOCO), using a mixture of white and brown for opacification of the substrate of Tooth 12. (o) Application of opaquer on the substrate of Tooth 12. (p) Palatal shell construction on Tooth 12 using Incisal composite resin (Forma, Ultradent). (q) Application of the dentin layer on Tooth 12 using A2D composite resin (Forma, Ultradent). (r) Application of the enamel layer on Tooth 12 using Transcend UB composite resin (Ultradent, United States). (s) Immediate final aspect of the patient's smile after the completion and polishing protocol with diamond-based polishers on composite resin veneers from Teeth 13 to 23.

Following periodontal surgery, orthodontic spacers were placed in the same clinical session to create diastemas at specific points, allowing aesthetic rehabilitation without causing occlusal impairment (Figure 8). After 7 days of postoperative recovery, the restorative phase began, using composite resin veneers from Teeth 13 to 23 to restore aesthetics and patient satisfaction (Figure 9).

**Figure 10:**
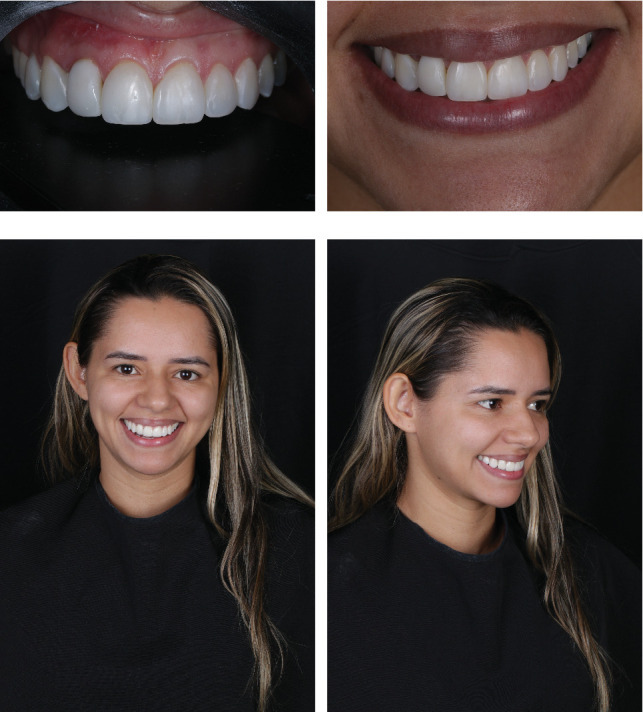
(a) Frontal view of the smile after 18 months. (b) Approximate extraoral photograph of the smile after 18 months. (c) Extraoral photograph of the smile after 18 months. (d) Extraoral lateral right view of the smile after 18 months.

**Figure 11:**
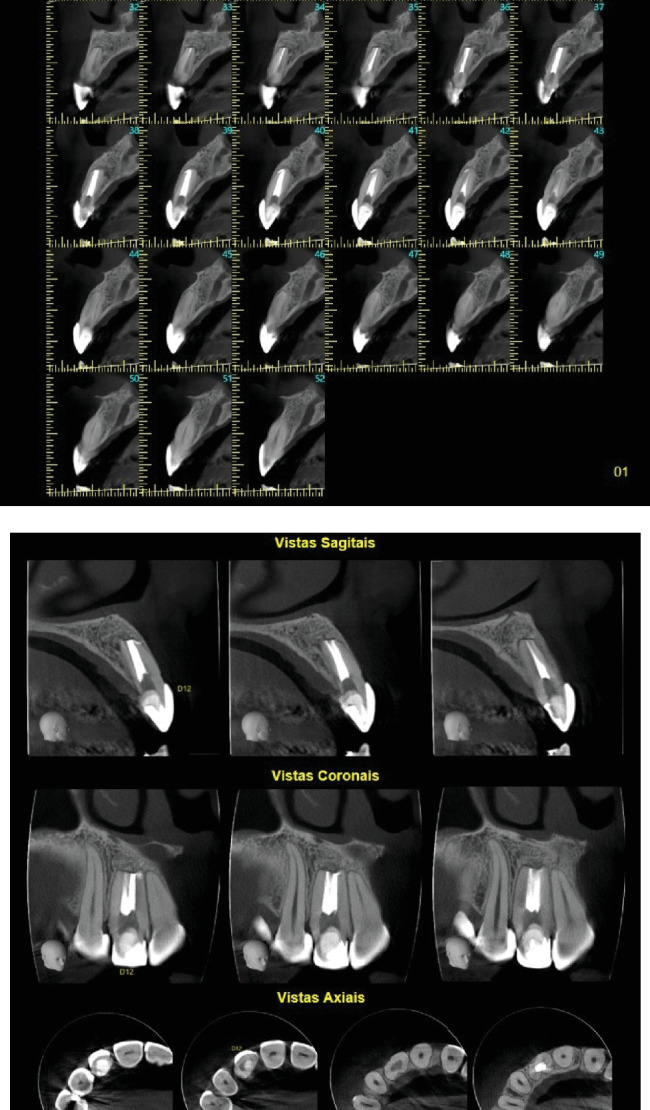
(a, b) Cone-beam computed tomography with small volume and high resolution, with axial, sagittal, coronal, and parasagittal cuts of 1-mm thickness and spacing of 0.5 mm at 0.5-mm intervals. Tooth 12 with satisfactory endodontic treatment shows a dental anomaly in shape. There is an observed flattening of the root apex, consistent with periapical surgery. The structure of the remaining alveolar bone tissue is normal, as is the density of the spongy bone.

Upon completion of the patient's treatment plan, the need for regular follow-up was instructed at 30, 90, and 180 days and return after 12 and 18 months. Following the protocol, after 1 year of treatment, clinical examination (Figure 10) and radiographic examination (Figure 11) demonstrate satisfaction with aesthetic rehabilitation and the success of surgical treatment. This underscores the importance of periodic maintenance of aesthetic rehabilitation and monitoring of surgical cases.

## 3. Discussion

This article demonstrates the importance of communication among specialties in resolving multidisciplinary cases. Dentistry requires a holistic approach that addresses patient needs, often extending beyond a single area. Therefore, dialogue among specialties becomes essential to deliver results that encompass three pillars: health, function, and aesthetics. Multidisciplinary treatment is aimed at restoring patients to a state of health while returning their physiological functions. By recovering health and function, aesthetics naturally emerges as a result of collaborative work among dental specialties.

Nonsurgical endodontic treatment is a modality with high predictability; however, certain factors can lead to failures related to anatomical and/or technical difficulties, compromising the success of endodontic treatment and allowing additional interventions. In this context, endodontic retreatment can be performed surgically or nonsurgically. Surgical retreatment is recommended when attempts at nonsurgical retreatment fail or are considered impractical. Before deciding on surgical retreat, a thorough evaluation of the etiology of persistent pathology is essential [13].

Various factors related to the tooth may require surgical endodontic retreatment, including the presence of complex root canal anatomy, bifurcations in the middle and apical thirds, and modification of root anatomy due to previous endodontic treatment [13]. All these factors, individually or in combination, can lead to ineffective biomechanical disinfection [14]. Therefore, the contemporary approach to surgical endodontic therapy employs advanced techniques such as the use of high magnification and illumination through surgical microscopes, ultrasonic instrumentation, and biocompatible filling materials, resulting in high efficacy in treating complex cases [15, 16].

The based in the treatment strategy of similar cases published in the literature, a multidisciplinary nature of the case, demonstrates the relationship between the main endodontic, oral and maxillofacial, periodontal, and restorative approaches [3, 17, 18]. It illustrates the success in diagnosis, treatment, and regenerative prognosis by performing endodontic treatment followed by the use of the dermal acellular matrix [17]. With the success of the procedure, it is possible to begin the aesthetic procedure in a necessarily invasive and individualized manner for the patient [3].

The association between periodontics and dentistry is crucial to the aesthetics of the new smile. The integration of gingivoplasty and composite resin veneers, when combined, restores the patient's self-esteem and quality of life [17, 18].

Composite resins are classified on the basis of particle size: microparticulate, hybrid, and nanohybrid. Current resin materials possess high mechanical properties; however, exposure to high-stress situations such as bruxism or parafunctional habits increases the risk of fracture and wear, as anything that wears down the tooth structure also wears down the restorative material. Additionally, maintaining gloss and polish for resin durability is a significant challenge, requiring periodic maintenance and follow-up due to various factors such as the patient's diet and lifestyle. Therefore, maintenance is individualized according to host habits and should be performed regularly to increase the success of the rehabilitation [3, 19–21].

From the authors' point of view, this case employed a minimally invasive strategy, avoiding veneers on all anterior teeth and avoiding post and core on Tooth 12, thus presenting as a low-cost option. However, maintaining the quality of the composite resin will require the patient's commitment to the follow-up schedule. Therefore, the patient should follow a 6-month maintenance plan involving resin repolishing and possible repairs to ensure the continued success of the treatment.

## 4. Conclusion

This report demonstrates to the dentist a safe multidisciplinary approach, grounded in scientific evidence, and following a treatment plan to the patient's needs. With clinical and radiographic monitoring for 18 months, the success of oral rehabilitation involving endodontics, bucco-maxillo-facial surgery, periodontics, and dentistry is evident, providing function, aesthetic satisfaction, and quality of life for the patient.

## Data Availability

All data related to the presented case are included in this published article.
